# Evaluation of the EAWS Ergonomic Analysis on the Assembly Line: Xsens vs. Manual Expert Method—A Case Study

**DOI:** 10.3390/s25154564

**Published:** 2025-07-23

**Authors:** Matic Breznik, Borut Buchmeister, Nataša Vujica Herzog

**Affiliations:** 1 BSH Nazarje, Savinjska cesta 30, 3331 Nazarje, Slovenia; matic.breznik@bshg.com; 2 Faculty of Mechanical Engineering, University of Maribor, Smetanova ulica 17, 2000 Maribor, Slovenia; borut.buchmeister@um.si

**Keywords:** Xsens, motion capture, EAWS, ergonomic analysis, reliability

## Abstract

**Highlights:**

**What are the main findings?**

- Strong similarity found in whole-body EAWS ergonomic assessment results between Xsens technology with Process Simulate V16 software and expert evaluations.
- Significant discrepancies observed in upper limb analysis, indicating areas where the Xsens system requires further validation

**What is the implication of the main finding?**

- Xsens motion capture technology shows the potential of motion capture to improve assessment efficiency and accuracy, supporting safer and more productive work environments.
- Further analyses with a wider range of participants are needed to confirm the present results.

**Abstract:**

This study investigates the effectiveness of the Xsens motion capture system in performing ergonomic analysis compared to traditional manual assessments by experts in the specific environment of assembly lines. A comprehensive literature review emphasizes the need to investigate the reliability of new, promising high-tech systems. The main objective was therefore to compare the Ergonomic Assessment Worksheet (EAWS) assessment approach performed with Xsens motion capture technology and Process Simulate V16 software with the manual method using EAWS digital prepared by experts in the controlled workflow. The greatest value of the research conducted lies in the novel integration of the state-of-the-art Xsens motion capture technology with the Process Simulate V16 software environment and the use of the licensed EAWS ergonomic method and Methods-Time Measurement Universal Analyzing System (MTM-UAS). The results are presented in the form of a case study. The results show a large similarity between the whole-body results and a large difference in the upper limb results, confirming the initial benefits of the Xsens equipment but also pointing to the need to verify its reliability on larger samples. The study highlights the potential of integrating Xsens motion capture data into ergonomic assessments and tuning of the assembly line to increase productivity and worker safety.

## 1. Introduction

In this study, we investigate the effectiveness of Xsens [1] inertial measurement units (IMUs) and motion capture systems in performing ergonomic analyses compared to traditional manual assessments by on-site experts. The study investigates the application of motion capture technology in industrial environments with assembly lines, similar to previous studies that have used Xsens to assess ergonomic risks in other environments [2], but in our case, we also used additional software for EAWS analysis with Process Simulate V16. Assembly lines are a typical production environment where ergonomic approaches can provide tangible and measurable results. In these environments, where workers often perform repetitive tasks, even small errors or delays can lead to a series of problems. With proper ergonomic workplace design, companies can achieve shorter cycle times, higher productivity, lower production costs, better product quality, fewer human and system errors, and fewer injuries and injury-related expenses.

Assembly work is one of the most common activities in industrialized countries. Particularly in automated industries such as the automotive, steel, and electrical industries, employees are often exposed to high levels of physical strain. In highly industrialized countries, almost a third of all sick leave is due to musculoskeletal problems, which can be caused by poorly designed ergonomic workplaces. Various methods are available for assessing physical ergonomics in industry, e.g., OWAS, RULA, REBA, NIOSH, OCRA, LUBA, ERIN, EAWS, and many others presented by [3], but only a limited number of these are available in computerized tools (e.g., Siemens Jack and Process Simulate V16).

For our study, the EAWS method was selected, which was developed specifically for evaluating the movements of workers on assembly lines. The manual implementation of the EAWS requires specially trained labor analysts, while a special license is required to use the EAWS method with software packages. For our research, we used the Process Simulate V16 software package, which does not include the EAWS for free use but can be activated by purchasing a license.

The feasibility of using IMU-based motion capture for ergonomic assessment has also been supported by other research efforts. For instance, ref. [4] demonstrated the potential for automated EAWS scoring using deep learning models. Similarly, ref. [5,6,7] validated the approach based on IMU data and expert labeling, reinforcing the broader applicability of sensor-based ergonomic evaluation methods.

The research was made possible by the involvement of specially trained labor analysts and the use of sophisticated equipment, including the Process Simulate V16 software environment with the licensed EAWS method, the Xsens suit, and a motion capture system. As previously described in [8,9], motion capture technology is becoming a cornerstone for accurate and objective ergonomic assessments, but there is still a lack of research confirming its reliability. The main objective of our research was therefore to compare the results of two ergonomic assessment approaches: the EAWS analysis performed with a motion capture system and the assessment performed by a certified expert. A controlled work sequence was designed and performed at a selected workplace with a specific worker. Using controlled laboratory recordings and expert assessments, we investigated the influence of motion capture systems on the accuracy and consistency of ergonomic analyses. The results are highly insightful and have the potential to improve ergonomic assessment methods.

The literature reviews also highlighted another problem: the lack of large-scale data or evidence on the reliability of the results obtained with various high-tech methods of capturing and analyzing movement with different sensor systems. Despite advances in the use of motion capture systems for ergonomic assessments, the literature shows a gap in comprehensive validation studies that evaluate the repeatability and reliability of these systems compared to traditional methods in industry. Therefore, we decided to invite more certified experts to our study and address this topic as well. In the end, only five of them performed the analysis and presented the results, so we decided to present the collected results as preliminary to encourage further research of this kind. The problem is that the number of certified EAWS experts is very limited.

The incorporation of ergonomic considerations into the assembly line process has been widely studied, particularly in relation to factors such as productivity, efficiency, and worker well-being, with a focus on reducing musculoskeletal risks and optimizing worker health [10,11]. Several conventional ergonomic tools such as Rapid Upper Limb Assessment (RULA), Rapid Entire Body Assessment (REBA), and the EAWS have been developed to assess and address these risks [12]. However, despite their widespread use, these tools are highly dependent on subjective assessments and manual data collection, which can lead to variability in results due to assessor bias or inconsistency [11,13]. This variability emphasizes the importance of developing more objective and repeatable assessment methods.

Among the advances in simulation technologies for ergonomic assessment, the use of Siemens’ Process Simulate V16 software was highlighted along the EAWS. A case study by [14] demonstrated the integration of these tools to assess ergonomic risks in the design phase of production processes. With the help of Process Simulate V16, which enables the creation of a digital human model, they were able to virtually simulate assembly tasks and proactively initiate corrective measures. The integration of the EAWS supports this framework by providing a structured and internationally recognized approach to the standardized assessment of ergonomic overload.

As an alternative to traditional methods, recent advances in motion capture technology, particularly inertial sensor-based systems, provide more accurate and repeatable data for ergonomic assessments that overcome the limitations of manual and subjective assessment. Ref. [15] highlights the importance of transparency of inertial sensor technologies for ergonomic assessments, as the transparency of these systems can influence their effectiveness and user confidence, which is a crucial factor for the success of these systems.

Advances in motion capture technology have produced systems that provide accurate, objective data on body movements and significantly reduce reliance on human interpretation. Studies have demonstrated the effectiveness of Xsens in various applications, such as high-risk occupations and industrial environments where ergonomic risks are prevalent [2,16,17,18,19]. By providing detailed data, Xsens enables targeted ergonomic interventions and the optimization of work processes.

Validation of an ergonomic assessment method using Kinect data in real workplace conditions [20] also highlights the growing role of advanced technology in ergonomics. The study supports the notion that Kinect-based systems provide reliable ergonomic assessments, offering significant advantages over traditional methods in terms of reliability.

The study conducted by [21,22] shows that while Xsens provides an acceptable level of accuracy, the complexity of the task can lead to errors, especially when tracking fine joint movements.

High accuracy and reliability in ergonomic risk assessment have previously been researched for IMU-based systems by [23,24]. The sensors recorded data at a sampling rate of five frames per second. The reason for the low frames per second is the fact that ergonomic analysis did not provide the same quality of results with the higher frames. The reason for this is that several positions were created in the EAWS, which took 0.1–0.2 s and negatively affected the final result. The option of lowering the frame rate was also confirmed by a study stating that sampling rates above 3 frames/second were not significantly different from the cumulative loads obtained at 60 frames/second [25].

Ref. [26] demonstrated the real-time application of motion capture technology for ergonomic risk assessments in dynamic work environments, particularly focusing on postural ergonomics. Their study showed how motion analysis systems can effectively capture body movements and identify awkward postures that can lead to increased musculoskeletal risk. Although this study focused on surgeons, the methodology and results are highly relevant to a wide range of industries where people perform physically demanding tasks with repetitive movements. The ability to assess ergonomic risks using motion capture systems provides valuable data for preventive measures and workplace adjustments that can significantly improve the health and performance of workers in any field. This approach is in line with the growing trend of using motion capture and digital tools to automate ergonomic assessments. While the rise of Industry 4.0 technologies, tools such as virtual simulations and augmented reality have been introduced to improve manufacturing efficiency and worker safety [27]. Motion capture systems are increasingly recognized as indispensable tools in this context. Studies [8,27] have emphasized the potential of motion capture systems for the automatic assessment of workplace ergonomics, enabling real-time feedback. Research emphasizes their ability to integrate seamlessly with digital twins and virtual environments to provide a comprehensive overview of ergonomic challenges and enable targeted interventions [28,29]. Ref. [15] discusses how the integration of inertial sensors with such technologies must be transparent to ensure that users can trust the results and make data-driven ergonomic decisions effectively.

Despite advances in the use of motion capture systems for ergonomic assessments, the literature shows a gap in comprehensive validation studies assessing the repeatability and reliability of these systems compared to traditional methods in industry.

## 2. Research Methodology

A case study approach was selected to provide a detailed and focused evaluation of the Xsens system in a realistic industrial setting. While the use of a single subject limits the generalizability of findings, it enables close control of experimental conditions and allows for a direct comparison with expert evaluations. Future studies will be required to verify these results across a broader participant base.

### 2.1. Research Framework

**Figure 1:**
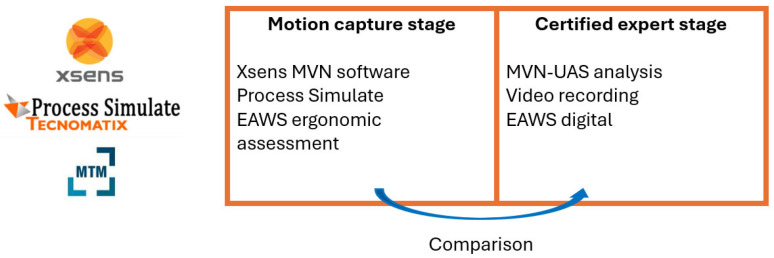
Research framework.

The aim of our research was to compare the results of the EAWS ergonomic analysis obtained using a motion capture system and the analysis performed by a certified expert. The research framework is shown in Figure 1. A controlled work sequence was designed and carried out at a selected workplace with a selected worker.

The Xsens sensor suit [30] with 17 wireless motion trackers is one of the latest devices for motion detection. It can be used to record workers’ movements, which can later be analyzed using various ergonomic approaches. The motion trackers measure 3D angular velocity, 3D acceleration, the earth’s magnetic field, and atmospheric pressure. In combination with the Xsens algorithms, these measurements ensure drift-free 3D orientation. Sensor calibration is required before starting the tracking process.

The MVN 2024.2 Analysis Software Engine was used to record sensor data. This software integrates data from individual motion trackers with a biomechanical model of the human body to determine segment positions and orientations.

Process Simulate V16 [31] was used to model the workplace, simulate worker movements and perform ergonomic analysis. Process Simulate is a digital manufacturing software developed by Siemens PLM Software V16 that enables the simulation, validation, and optimization of manufacturing processes in a virtual environment. It is used in industries such as automotive, aerospace, and electronics to improve production efficiency. The software allows users to create 3D simulations of assembly lines, robotic processes, and human interactions with machines, identifying potential design flaws, safety risks, and inefficiencies before physical implementation. For our research, Process Simulate V16 software was used to conduct an ergonomic assessment with the EAWS method.

The Ergonomic Assessment Worksheet (EAWS) was used to evaluate physical workload based on posture, manual handling, and repetitive tasks, following its standardized scoring protocol. In this study, the EAWS was applied using motion data from Xsens and video-based expert assessments. For detailed methodological background, readers are referred to the official EAWS documentation [32]. If ergonomic risks are identified, recommendations for improvement may include redesigning workstations, introducing lifting aids, changing work processes or training workers in safer work practices.

The MTM-UAS (Methods-Time Measurement Universal Analyzing System) [32] is a predefined motion time system for the analysis and optimization of manual work processes. It provides standardized time values for basic human movements, such as grasping, positioning, and releasing, enabling accurate work measurement without the need for direct time studies. The MTM-UAS is particularly useful in manufacturing and assembly environments where efficiency, ergonomics and productivity are important. By breaking down tasks into basic movements and assigning predetermined times, the MTM-UAS helps companies design optimized workflows, reduce unnecessary movements, and improve work performance. The system is widely used for workplace design, labor cost estimation, and ergonomic assessments, supporting lean manufacturing principles and improving overall operational efficiency.

A controlled workflow was designed and performed at a selected workstation with a selected worker.

### 2.2. Research Environment

The experiment was conducted in a company that specializes in the production of small household appliances and mainly performs assembly line work. These appliances include a variety of kitchen and household products for daily use. The workflow examined in this study included 14 standardized assembly operations that reflect typical tasks in such a factory environment. These tasks included precise assembly, accurate placement of small parts, and performing visual quality control to ensure compliance with manufacturing standards.

The nature of the components used in the workflow ensured that no heavy lifting or strenuous movements were required, as all parts weighed less than 1 kg. This setup reflected the ergonomic requirements common in the production of small domestic appliances.

**Figure 2:**
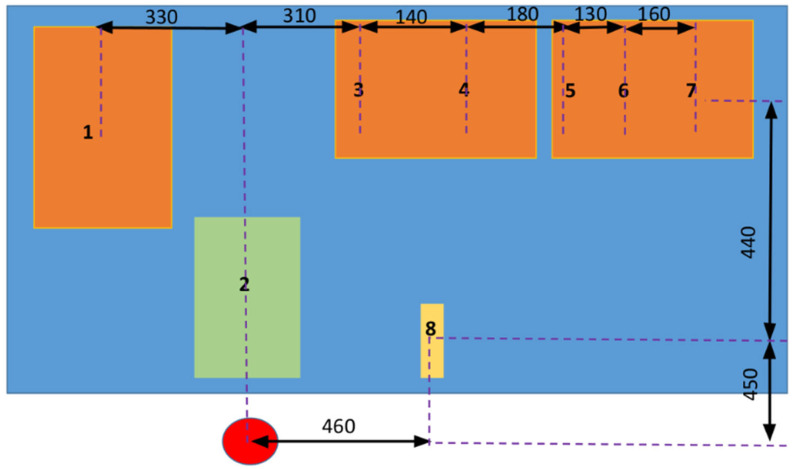
Layout representation of the workstation.

The design of the workstation was standardized. The height of the objects and the length of the worker’s reach were determined and measured in advance to ensure consistency between the repetitions of the experiments and the preparation of the expert analysis. The red dot represents the center of the worker’s chest. The distances shown in Figure 2 are in millimeters. The height of the workpiece (2) is 1000 mm, the height of the orange parts is 1200 mm, and the height of the yellow part is 700 mm. The detailed Methods-Time Measurement Universal Analyzing System (MTM-UAS) analysis of the workstation was specifically performed as input before the EAWS analysis. The process time is 21.96 s, and the specific times of the operations are listed in Table 1.

### 2.3. Procedure

#### 2.3.1. Motion Capture Stage

Xsens: The Xsens MVN 2024.2 Awinda motion capture system was used, consisting of 17 wireless inertial sensors (IMUs) placed on key body segments including the head, upper arms, forearms, hands, torso, pelvis, thighs, shins, and feet. Each sensor integrates a 3D accelerometer, gyroscope, and magnetometer. The system operated at a sampling rate of 60 Hz and transmitted data wirelessly to a base station. The sensors were attached using adjustable straps to ensure firm and repeatable placement on the subject’s body. The system was calibrated to ensure accurate motion tracking. Calibration involves a multi-step verification process in which the subject assumes standardized postures to match sensor readings with anatomical positions. The data were then recorded while the subject performed the predefined work sequence described in Table 1. The recording session consisted of 10 full repetitions of the assembly process, captured continuously during a single session.

**Figure 3:**
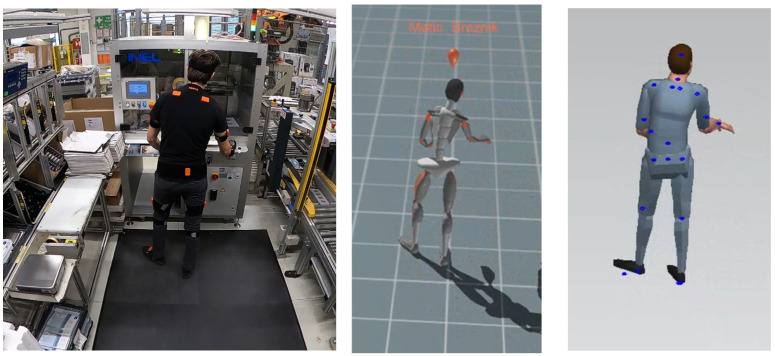
Experiment in progress. Motion capture recording phase.

Figure 3 shows the experiment in progress during a motion capture. After data collection, the motion data were analyzed using Xsens’s MVN 2024.2 Analyze software, which interprets the sensor readings and converts them into motion data to create a detailed motion profile of the worker. The importance of integrating human factors and ergonomics in Industry 4.0, which underlines the value of motion capture systems for optimizing occupational safety, has already been presented in [17,33,34,35], as well as in prior work by the authors focused on a similar industrial setting [36]. In addition to digitalization, personalization also offers a great advantage, as separate ergonomic analyzes can be created for several employees at the workstations. Differences in terms of gender, height, or age of the employees can be considered [37,38].

Process Simulate V16: The motion data recorded by Xsens were uploaded to Process Simulate V16, a digital manufacturing tool from Tecnomatix Siemens that enables the analysis and optimization of industrial tasks. In this step, the movements from the created digital twin [14] are segmented into individual work steps to map the individual parts of the work process. We have named the work steps so that we can later read the EAWS report and know exactly which part of the assembly process the report step is about. The segmented parts, which represent work steps, were then exported to EAWS digital.

**Figure 4:**
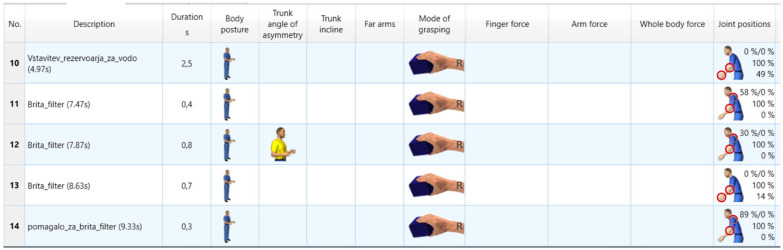
Visualization of an ergonomic analysis in EAWS digital, with categorized work steps and the corresponding postures.

EAWS digital: The motion data were reviewed to ensure that the assembly steps, postures, and joint positions identified by the Xsens system were accurate and suitable for ergonomic analysis, as shown in Figure 4. The system then automatically generated an ergonomic analysis report that provided detailed insights into potential ergonomic risks based on the motion data captured. Initially, joint positions were included in the EAWS analysis to provide a detailed assessment of ergonomic risks by recording joint angles and postures. However, during the analysis, it became apparent that inaccuracies in the recording of elbow and wrist joint angles led to significant variations. These inaccuracies were probably due to the complexity of the fine rotational movements, which require high sensitivity and precision. Therefore, a separate analysis was performed without the joint positions, focusing solely on the posture of the limbs and the body movements. This adjustment significantly improved the repeatability of the results.

#### 2.3.2. Certified Expert Level

The EAWS expert was selected from a company specializing in the production of small household appliances and working in the assembly line design and ergonomics department. This qualification requires a two-week intensive training program followed by six months of supervised practice. The number of possible test subjects is therefore very limited. To ensure an unbiased assessment, the expert conducted his assessments independently and without prior knowledge of the results generated by the Xsens Motion Capture System.

Video recording: One complete assembly cycle was recorded using a conventional video camera and used for the expert’s manual ergonomic assessment. This footage was used by a certified ergonomics expert to manually assess the subject’s ergonomic risk factors. Importantly, this cycle was one of the repetitions captured by the Xsens motion capture system, ensuring that both assessment methods were based on identical performance data for a valid comparison.

EAWS digital: After analyzing the video footage and the MTM-UAS analysis, the expert used EAWS digital to complete the individual steps of the ergonomic analysis. The most important ergonomic risk factors, such as unfavorable postures or excessive forces, were identified and entered into the system. The expert then created an ergonomics analysis report that summarized his findings and included ergonomic risk assessments.

## 3. Results

**Figure 5:**
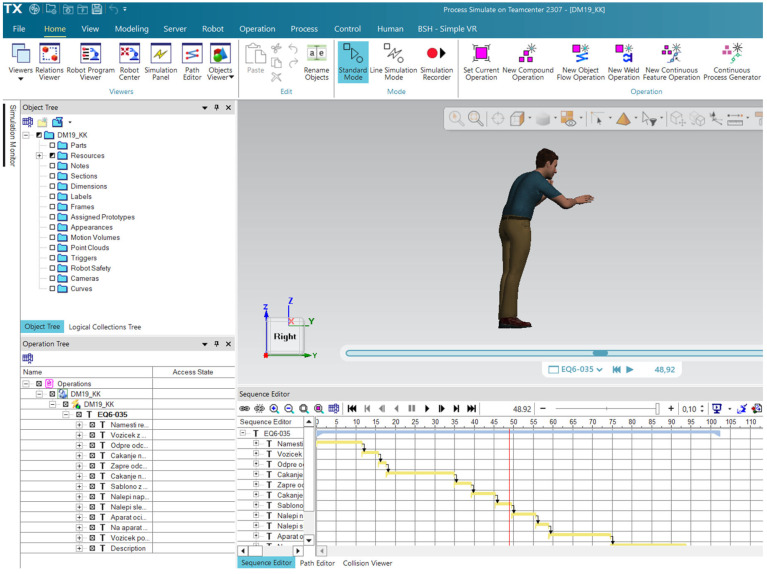
Step-by-step segmentation of operations in Process Simulate V16.

The motion capture session lasted approximately 25 s per recording, with each task performed in multiple repetitions to ensure consistency of the data. First, all operations are segmented, as shown in Figure 5, so that the process steps are clear. Then, once everything is complete, everything is prepared for export to EAWS digital to perform the ergonomic analysis.

**Figure 6:**
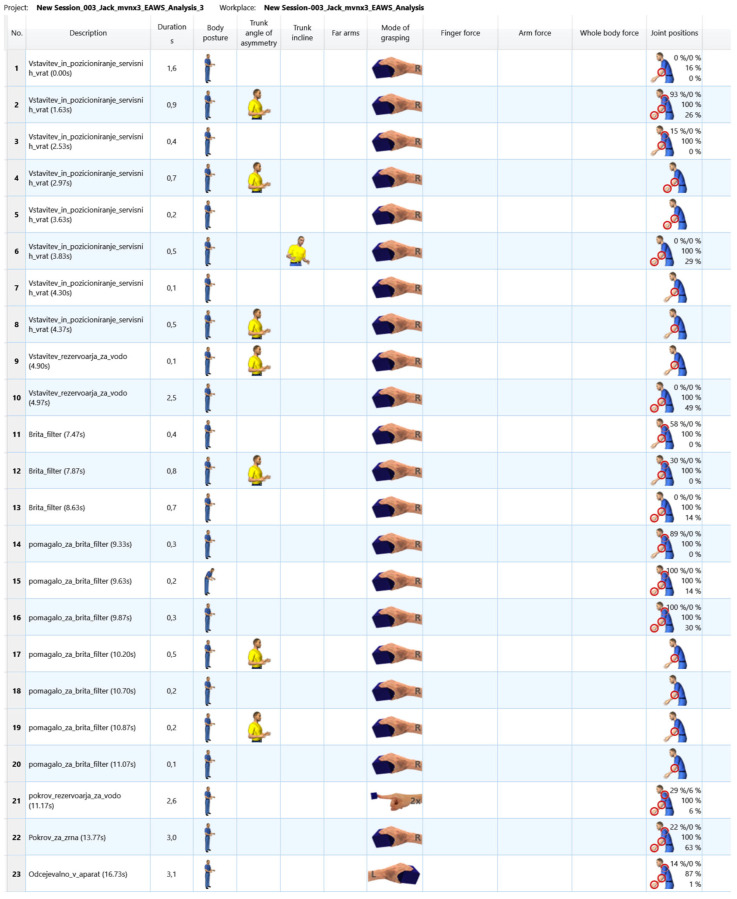
Results of the process steps performed with the Xsens Motion Capture data, including joint positions.

**Figure 7:**
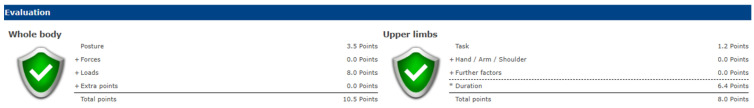
Result visualization example carried out with Xsens–Process Simulate V16–EAWS digital.

The results of the ergonomic analysis carried out with the Xsens Motion Capture data are presented in Table 2 and Figure 6 and Figure 7.

From the process steps shown in Figure 6, it can be seen that we have multiple repetitions of process steps that are less than 0.5 s. Each step added in the analysis has a grasping mode, although some of the steps in between did not involve holding an object. The decision that impacted the results the most was the severe evaluation of the joint positions, which are far too high and must be a fault of the system.

After we produced the initial results for the upper limbs with joint positions using Xsens, it became clear that the joint positions used in the analysis were inaccurate. In many process steps, the joint positions were defined at extreme angles that were clearly incorrect. This suggests that Xsens was struggling to accurately capture the rotations of the elbow and wrist. To address this issue, a subset of data labeled “without joint positions” was created to improve the consistency of the analysis.

**Figure 8:**
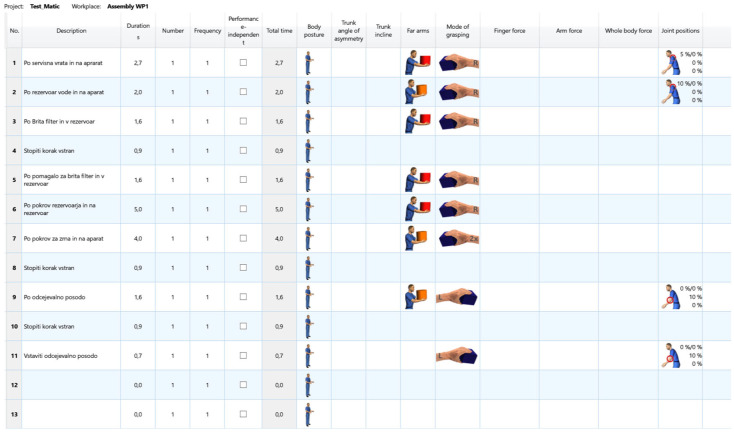
Results of the process steps performed by experts.

**Figure 9:**
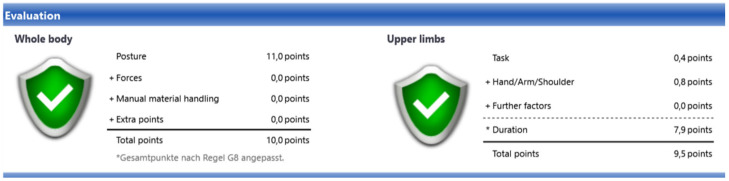
Result visualization example done with Video recording—EAWS digital.

To minimize the influence even further, we could exclude the process steps that take less than 0.5 s and try to add them to a subsequent process step, but we decided against this to keep the input data as unchanged as possible. The results of the ergonomic analysis, which was carried out manually by an expert on site using video recordings of the same work process, are shown in Table 3 and Figure 8 and Figure 9.

The number of process steps is almost halved when performed by the experts. The posture is stable. Torso angle and tilt are not as well defined as in the Xsens Motion Capture data. The arms are defined, but usually for too long of a time period, which leads to distorted final results. There are far fewer process steps with defined joint positions and those that are defined are much less extreme.

The reduction in the number of defined steps is largely due to the experts’ ability to group the movements into representative clusters and avoid over-segmentation. This is positive overall but can also have a negative effect if the steps are oversimplified.

The results show a great similarity between the whole-body results, but there is a big difference in the results for the upper limbs. To obtain more accurate results, a repeatability and correlation analysis should be performed.

## 4. Repeatability and Correlation Analysis

The literature review reveals a gap in the validation studies on the reliability and repeatability of high-tech motion analysis methods compared to traditional industrial methods. To address the problem of repeatability, our study was extended, including five more certified EAWS experts. Due to the small sample size, this is not a large-sample statistical analysis, but the results are still informative. The correlation and repeatability between Xsens and expert ratings was assessed using the Pearson coefficient correlation and the standard deviation of repeatability:

- The Pearson correlation coefficient measures the strength of the relationship between Xsens and the expert ratings, with values ranging from −1 (perfect negative correlation) to +1 (perfect positive correlation). The correlation coefficient was applied to upper limb and whole-body analyses, both with and without joint positions.
- The standard deviation of repeatability (SDR) quantified the variability in repeated measures and assessed the consistency of the Xsens and expert ratings across multiple trials. A lower SDR means higher repeatability.

Both methods were applied to multiple data sets, highlighting the performance of Xsens in motion analysis. The results of the ergonomic analysis performed with the Xsens motion capture system over ten recordings of the same workflow are shown in Table 4. Originally, the ergonomic analysis was performed including joint positions, but due to the high variability and inconsistent results, an additional analysis was performed without joint positions to improve the consistency of the results.

We invited several certified experts to participate in the study, but in the end only five of them performed the analysis and produced the final results. The problem is that the number of certified experts is very limited. In manufacturing companies, they are mostly involved in the design of assembly lines, so every answer from them is very valuable and important. The results of the ergonomic analysis, which was carried out manually by five experts on site using video recordings of the same work process, are shown in Table 5.

While Xsens provided consistent results in all trials, the expert ratings showed greater variability, particularly in the upper limb ratings.

To quantify the agreement between the Xsens analysis and expert evaluations, Pearson correlation coefficients were calculated for each body region: upper limbs and the whole body. EAWS scores from both methods were averaged across repetitions and then compared for each region. In cases where the Xsens output returned constant values (e.g., in upper limb assessments without joint position data), the correlation could not be defined and was therefore marked accordingly. This approach allowed for a direct numerical comparison between manual and digital evaluations.

The Pearson correlation coefficient (PCC) was used to assess the relationship between Xsens and the expert ratings. The PCC was calculated for Xsens with and without joint positions compared to the expert ratings using five recordings. For the upper limbs with joint positions, the stepwise calculation yielded a correlation of 0.89, indicating a strong relationship. The results for all scenarios are summarized in Table 6.

To assess the repeatability of the Xsens and expert recordings, the standard deviation of repeatability (SDR) was calculated. The SDR is a measure of variability in repeated measurements, with lower values indicating better repeatability. The analysis was performed for all available data sets, which are listed in Table 7.

**Figure 10:**
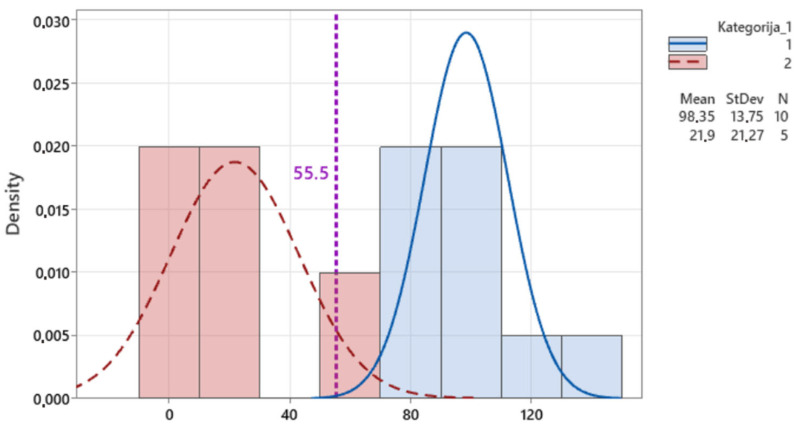
Upper limb results histogram.

**Figure 11:**
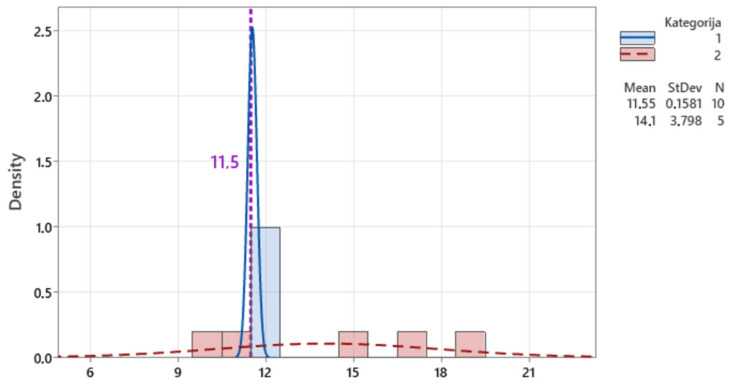
Whole body results histogram.

The results were also presented in the form of histograms with the distributions of the results of the ergonomic analysis in Figure 10 and Figure 11. The blue results represent the Xsens motion capture data with the joint positions, while the red results reflect the experts’ manual assessments. The purple line was added to represent the results of the Xsens motion capture data without joint positions.

## 5. Discussion

The aim of this study was to evaluate the effectiveness and reliability of motion capture systems, particularly the Xsens IMU-based system, for ergonomic assessments compared to traditional manual assessments by certified EAWS experts. The research context focused on a real industrial assembly environment, which provided a practical basis for evaluating the performance of these technologies under realistic conditions. The results reveal several important insights.

The results show that the two methods are very similar in the overall ergonomic assessment of the whole body. However, discrepancies were observed in the assessment of the upper limbs. Specifically, the Xsens system consistently resulted in higher severity ratings, primarily due to deviations in estimated joint angles. These deviations are most visible in the wrist and elbow, where subtle and complex movements are challenging to capture accurately using inertial measurement units (IMUs). Prior studies [21,22] have similarly reported that IMU-based systems may struggle with fine joint articulation due to limitations in sensor drift compensation, magnetic field interference, and biomechanical model assumptions. As a result, joint positions were excluded from some analyses in order to preserve result consistency and repeatability. Further investigations are needed to isolate and resolve the specific sources of this variability.

These discrepancies may be caused by several compounding factors: misalignment of sensors placed on small joints, insufficient resolution to capture rapid and fine-grained limb movements, and inertial drift over time. Furthermore, interference from metallic surroundings and magnetic fields common in industrial environments may degrade signal quality, leading to reduced tracking accuracy. The limited degrees of freedom in simplified biomechanical models can also introduce error when interpreting complex joint motions. As such, while Xsens shows strong potential for full-body ergonomic analysis, its use in evaluating upper limb activity requires careful interpretation, calibration improvements, and possibly integration with complementary sensing technologies.

To mitigate this problem, a second data set was created in which the joint positions were excluded, which improved consistency. However, even in this refined data set, short process steps under 0.5 s continued to have a negative influence on the result, suggesting that automated systems may need further calibration or post-processing algorithms to better group and interpret such micro-movements.

A key difference between the two methods was how the process steps were defined. The motion capture system produced a much higher number of segments as each movement was captured and categorized. In contrast, the expert demonstrated the ability to summarize micro-movements into more comprehensive, functionally significant actions, significantly reducing the number of steps defined. This simplification by the expert brings both benefits and challenges: while it minimizes over-segmentation, generalization can miss subtle risk factors present in micro-movements.

The study also highlighted the practical barriers to widespread adoption of automated EAWS assessments using Process Simulate V16 and motion capture technology. A separate license is required for the EAWS module, and certified experts are rare due to the specialized training required. These limitations highlight the need for more accessible tools and training programs for ergonomic assessments in the industry.

Our research aligns with recent findings [15] that emphasize the importance of transparency in sensor-based ergonomics systems. If users cannot trust the data due to inaccuracies in the detection of joint angles or excessive segmentation, the adoption of these tools in operational practice may be hindered. This highlights the need for ongoing validation studies and continuous user training to ensure that ergonomic decisions are truly data-driven and actionable.

Although the preliminary results are promising, the study also highlights the lack of comprehensive validation in terms of repeatability and inter-rater reliability. Future research needs to include repeated measures across various subjects and scenarios, as well as statistical correlation analyses between automated and manual assessments. This will be crucial to build confidence in the scalability and robustness of the technology.

## 6. Conclusions

In conclusion, our study provides valuable insights into the comparative performance of motion capture-based and expert-guided ergonomic assessments using the EAWS method in an industrial assembly environment. The integration of Xsens IMU data into Process Simulate V16 shows strong potential for streamlining and objectifying ergonomic assessments. However, current limitations, particularly in upper limb assessment and step segmentation, need to be addressed before widespread industrial application.

The results suggest that while motion capture systems can improve ergonomic analyses, particularly for trunk postures and general movement patterns, their use should be carefully calibrated and supplemented with expert supervision when assessing fine motor tasks. In addition, software accessibility, license requirements and the availability of trained experts remain barriers to implementation that require systemic solutions.

As only five certified experts took part in the comparison phase, these results must be regarded as provisional. Nevertheless, they provide a solid basis for future research and underline the need for larger validation studies. Bridging the gap between technological innovation and practical ergonomic assessment in industry remains a crucial goal, especially in the context of Industry 4.0, where real-time feedback and digital twins increasingly play a central role in optimizing both occupational safety and production performance.

Further research can follow two main directions. First, the reliability of the Xsens suit and mock-up system could be tested in a simplified, controlled environment with predefined motion sequences and a higher number of repetitions and with the use of multiple subjects. This would allow for a more robust statistical analysis, although the results may not be directly applicable to complex environments such as assembly lines.

Secondly, Xsens-based ergonomic analysis could be integrated into assembly line work. This would require the development of advanced algorithms that combine ergonomic data with time-based methods such as the MTM-UAS. The goal would be to design workflows that improve both efficiency and worker well-being. Although the accuracy of the system still needs to be improved, its high repeatability supports its use in balancing processes where consistency is important.

Another promising direction for future research involves a comparative evaluation of short-duration task segmentation strategies. Specifically, it would be useful to test the impact of excluding process steps below a certain time threshold (e.g., 0.5 s) and aggregating them into adjacent steps. Such an approach could help determine whether these refinements improve the accuracy and consistency of ergonomic assessments.

## Figures and Tables

**Table 1:** MTM-UAS time analysis results for the workstation.

Operation	Time [TMU]	Q × F	Total [TMU]	Total [s]
1	45	1 × 1.0	45	1.62
2	30	1 × 1.0	30	1.08
3	55	1 × 1.0	55	1.98
4	45	1 × 1.0	45	1.62
5	25	1 × 1.0	25	0.9
6	45	1 × 1.0	45	1.62
7	80	1 × 1.0	80	2.88
8	30	2 × 1.0	60	2.16
9	80	1 × 1.0	80	2.88
10	30	1 × 1.0	30	1.08
11	25	1 × 1.0	25	0.9
12	45	1 × 1.0	45	1.62
13	25	1 × 1.0	25	0.9
14	20	1 × 1.0	20	0.72

**Table 2:** Results of the ergonomic analysis, which were prepared based on motion capture recordings, recorded with Xsens.

Trial Number	Upper Limbs		Whole Body	
	With Joint Positions	Without Joint Positions	With Joint Positions	Without Joint Positions
1	88.5	55.5	11.5	11.5

**Table 3:** Results of the ergonomic analysis which were prepared based on expert manual analysis.

Trial Number	Upper Limbs	Whole Body
1	9.5	10.0

**Table 4:** Results of the ergonomic analysis, which were prepared based on motion capture recordings, recorded with Xsens—additional trials.

Trial Number	Upper Limbs		Whole Body	
	With Joint Positions	Without Joint Positions	With Joint Positions	Without Joint Positions
1	88.5	55.5	11.5	11.5
2	111	55.5	11.5	11.5
3	89.5	55.5	12	12
4	103	55.5	11.5	11.5
5	131.5	55.5	11.5	11.5
6	92	55.5	11.5	11.5
7	95	55.5	11.5	11.5
8	100	55.5	11.5	11.5
9	87	55.5	11.5	11.5
10	87	55.5	11.5	11.5

**Table 5:** Results of the ergonomic analysis, which were prepared based on expert manual analysis—added trials.

Trial Number	Upper Limbs	Whole Body
1	9.5	10.0
2	11.5	14.5
3	7.5	18.5
4	22.5	17.0
5	58.5	10.5

**Table 6:** Results of Pearson’s correlation coefficient for each scenario compared to the expert evaluation.

Scenario	PCC
Upper limbs with joint positions	0.89
Upper limbs without joint positions	Not defined due to constant values
Whole body with joint positions	0.65
Whole body without joint positions	0.65

**Table 7:** Results of the standard deviation of repeatability calculated for each scenario of ergonomic analysis.

Scenario	SDR	Average
Upper limbs with joint positions	14.01	98.45
Upper limbs without joint positions	0.00	55.5
Upper limbs experts	21.27	21.9
Whole body with joint positions	0.16	11.55
Whole body without joint positions	0.16	11.55
Whole body experts	3.80	14.1

## Data Availability

The original contributions presented in this study are included in the article. Further inquiries can be directed to the corresponding author(s).
